# The effects of mepiquat chloride on the lateral root initiation of cotton seedlings are associated with auxin and auxin-conjugate homeostasis

**DOI:** 10.1186/s12870-018-1599-4

**Published:** 2018-12-18

**Authors:** Xiaojiao Chen, Man Zhang, Mian Wang, Guiyu Tan, Mingcai Zhang, Yu Xia Hou, Baomin Wang, Zhaohu Li

**Affiliations:** 1 College of Agronomy and Biotechnology, China Agricultural University, Beijing, 100193 China; 2 College of Science, China Agricultural University, Beijing, 100193 China

**Keywords:** Mepiquat chloride, *Gossypium hirsutum* L., Lateral root initiation, IAA, IAA-amide conjugates, Amidohydrolase

## Abstract

**Background:**

Mepiquat chloride (MC) is a plant growth regulator widely used in cotton (*Gossypium hirsutum* L.) production to suppress excessive vegetative growth, increase root growth and avoid yield losses. To increase root growth, cotton seeds were treated with MC to increase the number of lateral root (LRs) and improve drought resistance. An increased indole-3-acetic acid (IAA) pool appeared to correlate with LR growth, and the principal source of IAA in germinating seeds is IAA conjugates. Here, the role of IAA homeostasis and signaling was investigated in cotton seedlings treated with MC.

**Results:**

In the present research, MC significantly increased endogenous IAA levels in the roots, which promoted lateral root initiation (LRI) by upregulating *GhARF7/19* and *GhLBD18*s and subsequently increasing LR quantity and elongation. The levels of IAA-amide conjugates significantly decreased in MC-treated seedlings compared with untreated control seedlings. Sixteen members of the cotton IAA amidohydrolase (IAH) gene family were identified, of which *GhIAR3a*, *GhIAR3b*, *GhILR1*, *GhILL3* and *GhILL6* were expressed during cotton seed germination. Compared with those in untreated control seedlings, the expression levels of *GhIAR3a*, *GhIAR3b*, *GhILR1* and *GhILL6* in the MC-treated seedlings were markedly elevated. The *GhIAR3a/b* and *GhILR1* genes were cloned and expressed in *Escherichia coli*; these recombinant proteins exhibited hydrolytic activity that could cleave IAA-phenyalanine (Phe), IAA-methionine (Met), IAA-glycine (Gly) and IAA-leucine (Leu) in vitro, while only GhIAR3a hydrolyzed IAA-alanine (Ala) efficiently. The content of GhIAR3a, as detected via an established sandwich enzyme-linked immunosorbent assay (ELISA), increased in the MC-treated seedlings compared with the untreated control seedlings. In addition, the Arabidopsis *iar3* mutant was less responsive to MC-induced LR growth than was wild type.

**Conclusions:**

These findings suggested that MC application could mediate IAA homeostasis via increased IAA levels from IAA-amide conjugate hydrolysis by accelerating IAH gene expression, which might promote LRI and increase the LR quantity and elongation.

**Electronic supplementary material:**

The online version of this article (10.1186/s12870-018-1599-4) contains supplementary material, which is available to authorized users.

## Background

The plant growth regulator mepiquat chloride (MC) has been used worldwide to suppress excessive vegetative growth, accelerate crop maturity and avoid yield losses in cotton production since 1975 [1–3]. In addition to manipulating the plant canopy structure, MC application to cotton seeds increases cotton root growth by increasing the number of lateral roots (LRs) and improves drought resistance [4–6]. Several studies have reported that MC is a gibberellic acid (GA) inhibitor and reduces cotton plant height by reducing endogenous GA levels via controlled GA biosynthetic and metabolic gene expression [7, 8].

GA inhibitors or deficiencies in GA interfere with endogenous levels not only of GAs but also of other plant hormones [7]. GA-deficient transgenic Populus exhibited increased LR density and length via interactions with indole-3-acetic acid (IAA) [9]. Mauriat showed that the application of paclobutrazol (a GA biosynthesis inhibitor) significantly increased the number of adventitious roots by enhancing IAA levels in the basal 3 mm of the stems of hybrid aspen cuttings [10]. It is well known that IAA is a pivotal regulator of LR growth in plants [11]. The root system is vital for plant development and is determined mainly by branching via LR formation [11, 12]. Therefore, we presumed that the promotion of LR growth by MC may be related to alterations in IAA levels.

IAA regulates LR development by inducing *auxin response factor* (*ARF*)*7* and *ARF19* expression, which activates downstream genes such as members of the *lateral organ boundaries-domain/asymmetric leaves2-like* (*LBD/ASL*) family [13–16]. Active, free IAA originates from de novo synthesis and IAA conjugate hydrolysis [17]. The latter is the principal source of IAA in germinating seed; for example, in maize and Arabidopsis (*Arabidopsis thaliana*), germination via the hydrolysis of conjugates stored in seeds rapidly provides free IAA to the developing seedling [18, 19]. Most IAA in plant tissues is conjugated at its carboxyl group to sugar moieties via ester bonds and amino acids or peptides via an amide linkage. Amide conjugates of IAA dominate over ester conjugates in dicotyledonous plants [20]. IAA-aspartic acid (Asp) and IAA-glutamic acid (Glu) are intermediates in IAA catabolism, while other conjugates, including IAA-alanine (Ala) and IAA-leucine (Leu), may act as reserve forms for the IAA homeostatic control of hormone concentrations [21].

IAA amidohydrolases (IAHs) release free IAA from their amide conjugates and thus are likely to play an important role in regulating active, free IAA levels in dicotyledonous plants [19]. Several IAHs (ILR1, IAR3, ILL1, ILL2, ILL3, ILL5 and ILL6) and their corresponding encoding genes, known collectively as the ILR1-like family, were initially characterized in Arabidopsis [22, 23]. The first isolated enzyme, ILR1, preferentially cleaves IAA-Leu and IAA-phenyalanine (Phe) in vitro, whereas the ILL1, ILL2, and IAR3 enzymes prefer IAA-Ala as a substrate; moreover, three additional IAHs show no activity on IAA-amide conjugates in Arabidopsis [24]. Active IAH orthologs have also been detected in other species, such as *Phaseolus vulgaris*, *Brassica rapa*, wheat and *Medicago truncatula* [25–28].

This study aimed to evaluate the roles of IAA metabolism and signaling in MC-induced increases in the number of LRs in cotton seedlings. It was hypothesized that cotton seed treatment with MC would increase the number of LRs by increasing IAA levels via the acceleration of IAA conjugate hydrolysis. Therefore, we determined the effects of MC on the levels of both endogenous IAA and IAA-amide conjugates and the expression patterns of genes encoding IAA signaling elements and IAHs during LR development. The genes encoding IAHs in cotton were cloned and functionally characterized in vitro. Rabbit polyclonal and mouse monoclonal antibodies (mAbs) against the IAH GhIAR3a were developed, and the effects of MC on GhIAR3a protein levels were detected via an established sandwich enzyme-linked immunosorbent assay (ELISA).

## Results

MC increased the number of LRs and lateral root primordia (LRP).

**Fig. 1:**
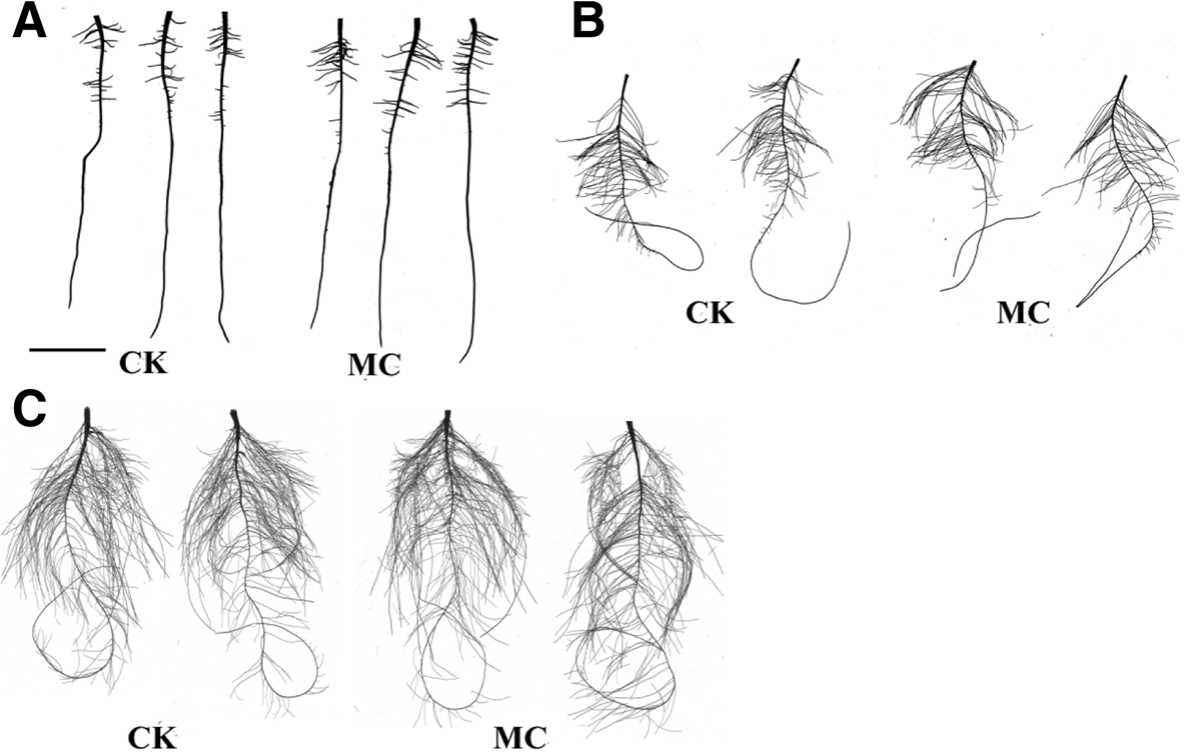
Effect of mepiquat chloride (MC) on lateral root (LR) development of cotton seedlings (**a**) to (**c**) Effect of MC on LR development of cotton seedlings at 6 (**a**), 12 (**b**) and 20 (**c**) d after treatment. Bars = 30 mm.

The LR number and length increased by 21.8 and 57.3%, respectively, at 6 days after MC treatment (dat) (Fig. 1a). Additionally, the LR number and length, root area, and root volume increased at 12 and 20 dat (Fig. 1b, c). This result indicated that, compared with the control, the MC treatment increased the root biomass and reduced aerial biomass, leading to a significant increase in the root-to-shoot ratio (Additional file 1: Figure S1).

**Fig. 2:**
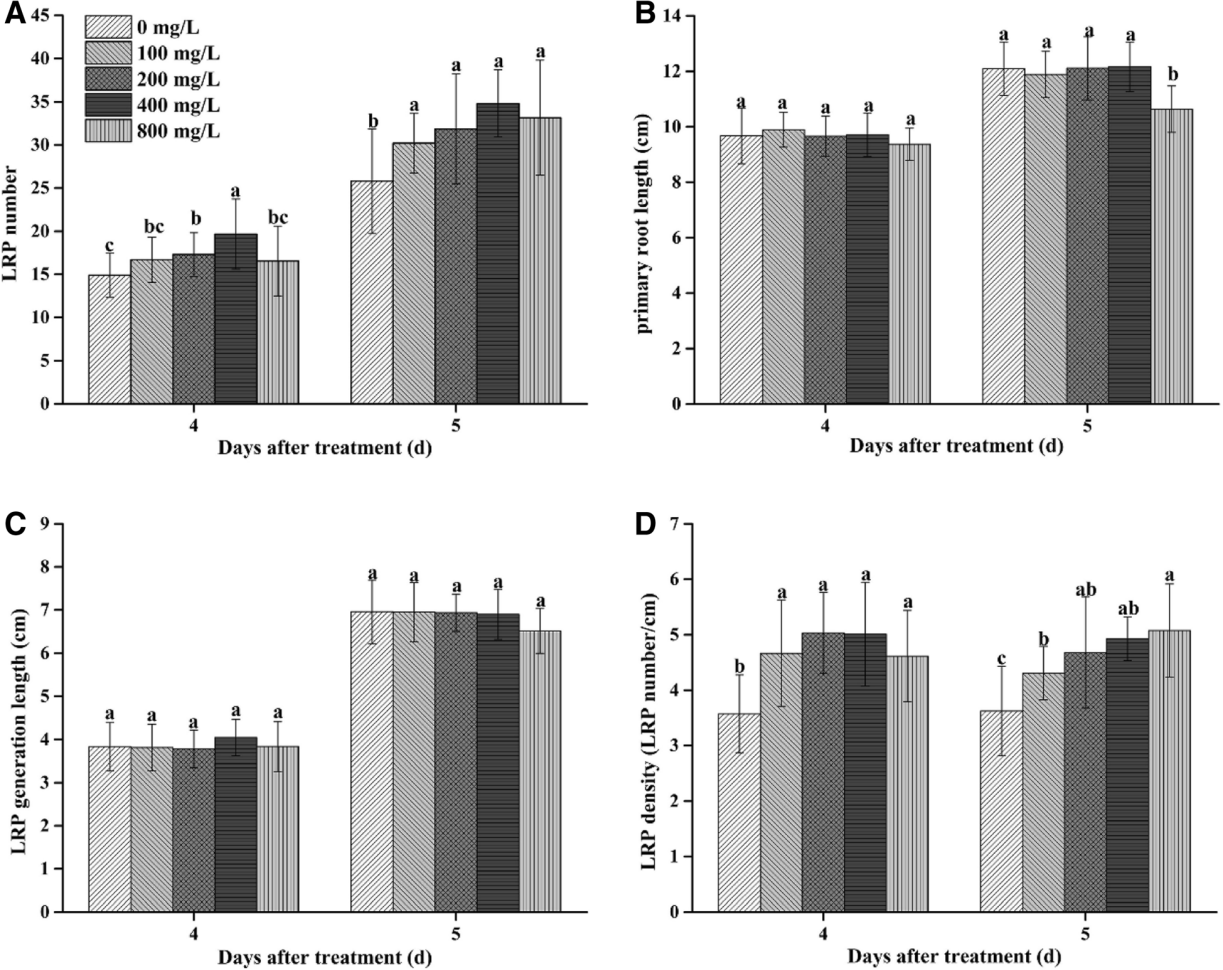
The effect of different concentrations of MC treatment on LR primordia (LRP) development of cotton seedlings. **a** the number of LRP, **b** length of primary root, **c** generation length of LRP and (D) density of LRP at 4 and 5d after MC treatment in cotton seedlings. Values are the means ± SD (*n* = 30); bars with the same letter are not significantly different at *P* < 0.05

The effects of different concentrations (0–400 mg/L) of MC on the number of LRP of cotton seedlings varied in a dose-dependent manner (Fig. 2a). At incremental concentrations from 100 to 800 mg/L, the number of LRP increased by 12.1 to 32.1% and by 17.1 to 34.9% at 4 and 5 dat, respectively. The effect was most pronounced at a concentration of 400 mg/L (Fig. 2a). In contrast, the primary root length of seedlings treated with 100 to 400 mg/L MC was unaffected (Fig. 2b). The MC treatment did not significantly alter the generation length of LRP (Fig. 2c). Thus, compared with that of the control plants, the density of LRP of the MC-treated plants increased (Fig. 2d). The time course of 400 mg/L MC’s action on LR formation showed that the number of LRP or LR increased by 32.1, 34.9, 21.8, 11.0 and 7.7% in MC-treated plants at 4, 5, 6, 12 and 20 dat, respectively. These data revealed that LR development was promoted by the MC treatment during early seedling development by increasing the number of LRP.

### MC accelerated the initiation of LRs

**Fig. 3:**
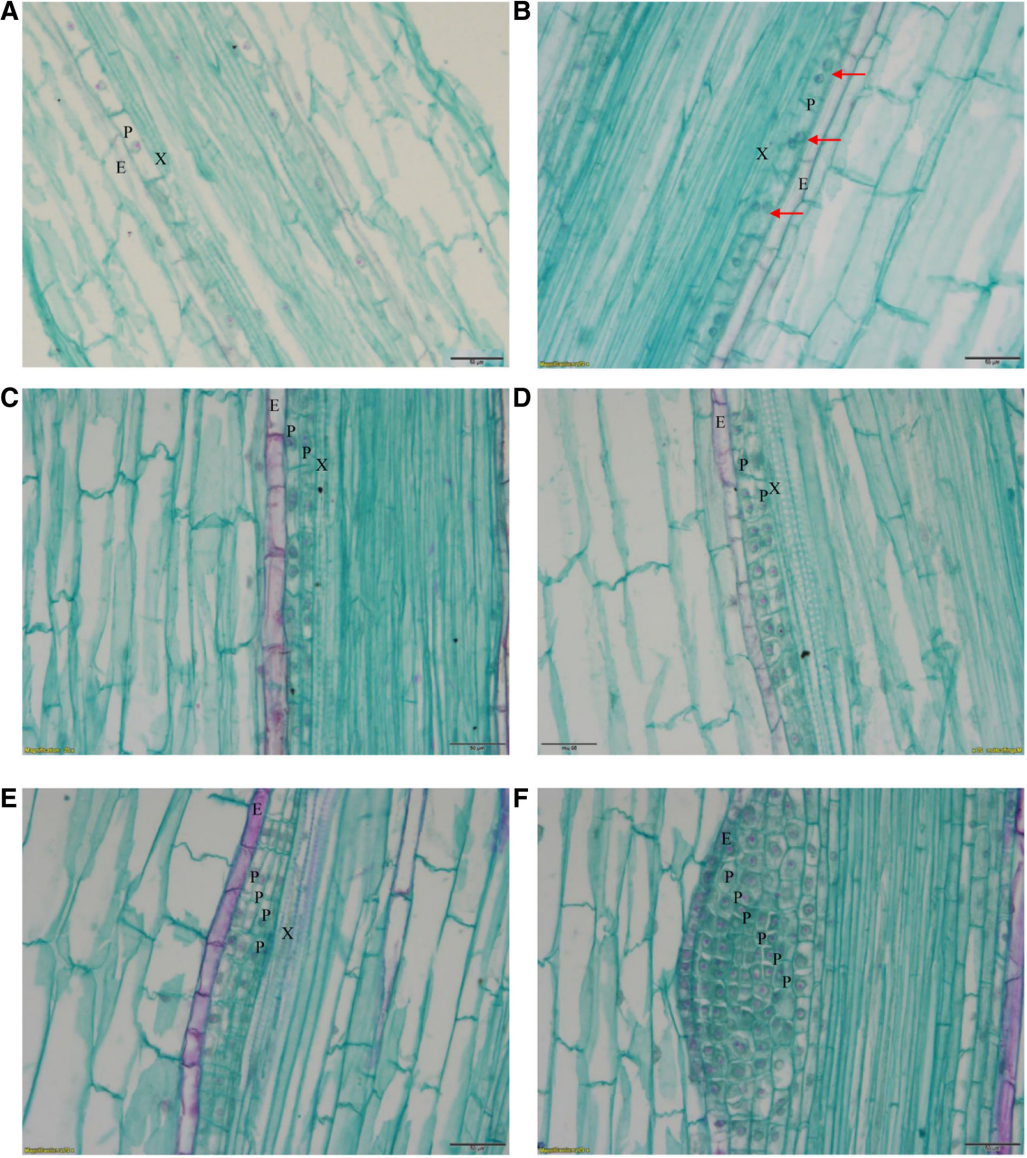
The effect of MC on morphology of the LRP in cotton roots. **a** to **b** Longitudinal sections of roots from the region 1.5–2.0 cm behind the root tip in control (**a**) and MC-treated (**b**) seedlings at 42 h after treatment. The LR initiation was observed in MC-treated seedlings. **c** to **d** Morphology of the LRP from the region 0.5–1.0 cm behind the root base at 2 and 3 d after treatment in control (**c**, **e**) and MC-treated (**d**, **f**) seedlings. Bar = 50 μm. E: endoderm; P, pericycle; X, xylem. The arrow points to the nucleus of cell dividing

To gain insight into the initial position of LRP, morphological changes of the MC-treated seedling were studied via paraffin sectioning. Periclinal division was observed in pericycle cells adjacent to a protoxylem pole approximately 1.5–2.0 cm behind the root tip, and the lateral root initiation (LRI) occurred earlier in the MC-treated seedlings than in the control seedlings (Fig. 3a, b). The priming of pericycle cells for LRI occurs behind the apical meristem [29]. The length of the root cap and meristem region was 332 ± 58 μM and 2574 ± 386 μM, respectively. The LRI priming region was approximately 0.3–1.5 cm behind the root tips of the cotton seedlings.

The LR development occurred sooner in the MC-treated cotton seedlings than in the control seedlings. The LRP gave rise to two cell layers at 2 dat in the MC-treated seedlings, while the LRP were still undergoing periclinal division in the control seedlings (Fig. 3c, d). Furthermore, there were 5.5 layers of LRP cells on average in the MC-treated seedlings at 3 dat, while there were only 4.1 layers on average in the control seedlings (Fig. 3e, f).

### MC increased IAA levels in cotton roots

**Fig. 4:**
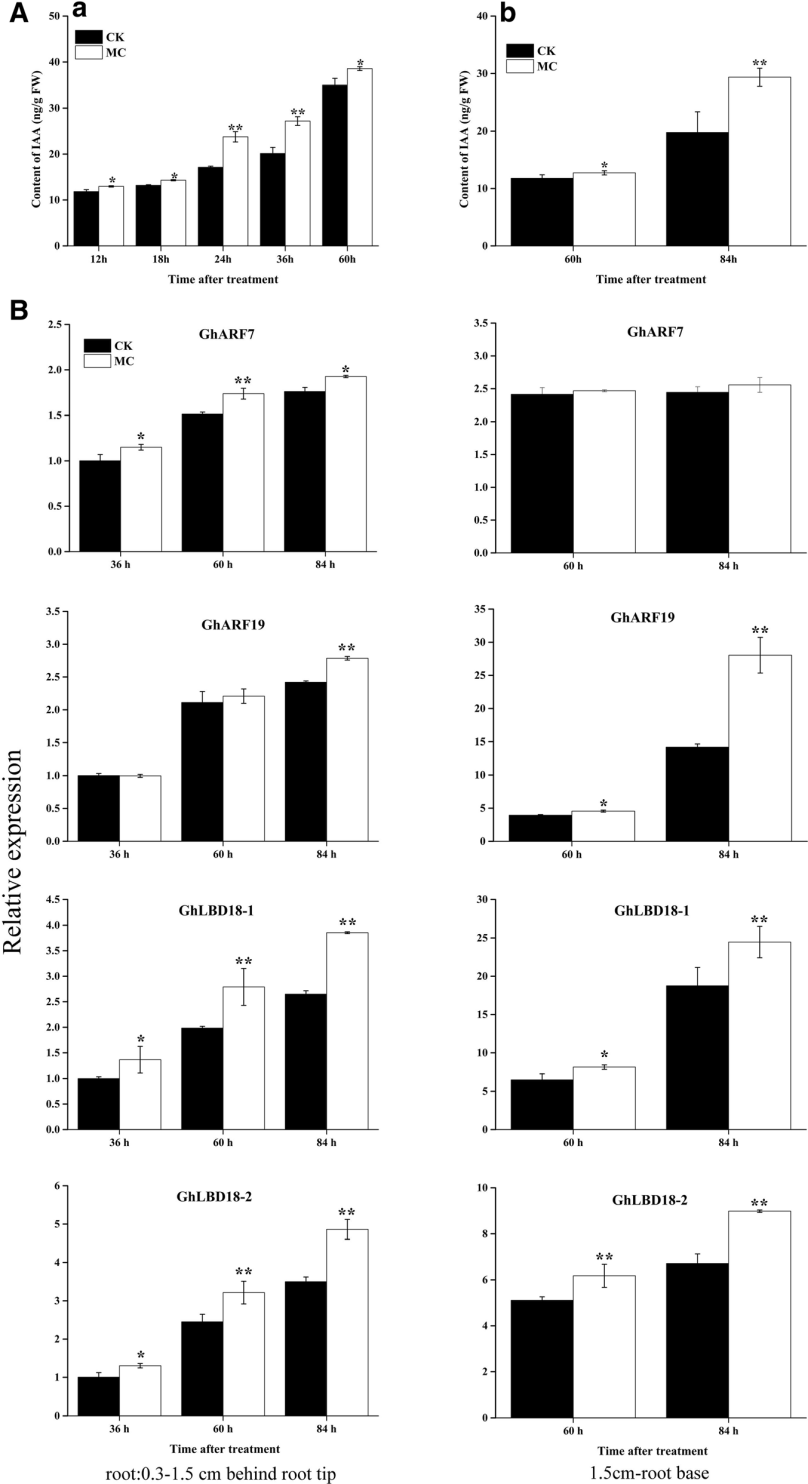
Effect of MC treatment on content of IAA and IAA signaling gene expression pattern (**a**) (a) Effect of MC on content of IAA in cotton seedlings. (**b**) Effect of MC on content of IAA in basal meristem of cotton roots (0.3–1.5 cm behind root tip). (**b**) The effect of MC on expression of auxin-signaling genes *GhARF7*, *GhARF19* and *GhLBD18s* during LR initiation in cotton roots. Values are the means ± SD (*n* = 4). Asterisks (*) and (**) indicate a significant difference (*P* < 0.05) and (*P* < 0.01), respectively, in comparison with the corresponding control.

There was significant increase in IAA levels in the LRI region of cotton roots in response to MC treatment. The IAA levels increased by 12.7 and 37.5% in the LRI region of cotton roots at 60 and 84 h after MC treatment, respectively (Fig. 4a b). The location of IAA accumulation plays a critical role in LRP priming for initiation [29]. Thus, we studied the distribution of IAA in the LRI priming region at 84 h after MC treatment. Using a mAb against IAA, we found a greater accumulation of IAA in the stele of LRI priming regions in the MC-treated group than in the control group (Additional file 1: Figure S2).

### MC upregulated the expression levels of *GhARF7*, *GhARF19* and *GhLBD18*s

LRI was under the control of *ARF7* and *ARF19* signaling modules. *LBD16* and *LBD18* are direct targets of *ARF7* and *ARF19*, both of which positively regulate LR formation during LRI [14]. The expression levels of *GhARF7*, *GhARF19*, *GhLBD18–1* and *GhLBD18–2* were upregulated in cotton roots at 36–84 h after MC treatment (Fig. 4b).

### MC affected IAA and IAA-amide conjugate levels

Dynamic changes in IAA and IAA-amide conjugate levels in the cotton seedlings were monitored after MC treatment. The IAA content significantly increased by 3.5–38.7% in the cotton seedlings at 12–60 h after treatment with MC (Fig. 4 a). The contents of 6 IAA-amide conjugates (IAA-Phe, IAA-methionine (Met), IAA-Ala, IAA-glycine (Gly), IAA-Leu, and IAA-isoleucine (Ile)) in the cotton seedlings were measured. Among those conjugates, the content of IAA-Phe was greatest, followed by that of IAA-Met, IAA-Ala and IAA-Gly; IAA-Leu and IAA-Ile presented the lowest contents, which were less than 0.1 ng/g fresh weight (FW), in the cotton seedlings (Table 1). Remarkably, the contents of IAA-Phe, IAA-Met and IAA-Ala decreased by 9.8–46.4%, 7.3–32.5% and 4.6–45.8%, respectively, in MC-treated seedlings at 3–60 h after treatment (Table 1).

**Table 1:** The effect of MC on the content of IAA-amide conjugates

Time after treatment (h)	IAA-Phe (ng/g FW)		IAA-Met (ng/g FW)		IAA-Ala (ng/g FW)		IAA-Gly (ng/g FW)	
	CK	MC	CK	MC	CK	MC	CK	MC
3	9.62 ±0.14	9.59 ±0.35	8.39 ±0.42	7.78 ±0.24	3.59 ±0.24	2.93 ±0.27^*^	1.02 ±0.13	1.20 ±0.33
6	8.43 ±0.33	7.60 ±0.32^*^	4.97 ±0.21	3.88 ±0.36^*^	3.06 ±0.32	2.92 ±0.13	1.14 ±0.13	0.92 ±0.25
12	7.41 ±0.53	5.44 ±0.25^*^	4.32 ±0.33	3.63 ±0.25^*^	2.89 ±0.10	2.70 ±0.14	1.40 ±0.14	0.97 ±0.52
18	6.34 ±0.36	3.64 ±0.35^*^	3.14±0.14	2.12±0.24^*^	2.10 ±0.11	1.14 ±0.13^*^	ND	ND
24	5.08 ±0.33	2.72 ±0.24^*^	ND	ND	ND	ND	ND	ND
60	3.03 ±0.35	2.43 ±0.35^*^	ND	ND	ND	ND	ND	ND

ND: not detected; Asterisks (*) indicate a significant difference (*P* < 0.05) in comparison with the corresponding control

### MC modulated the expression of cotton IAH genes

**Fig. 5:**
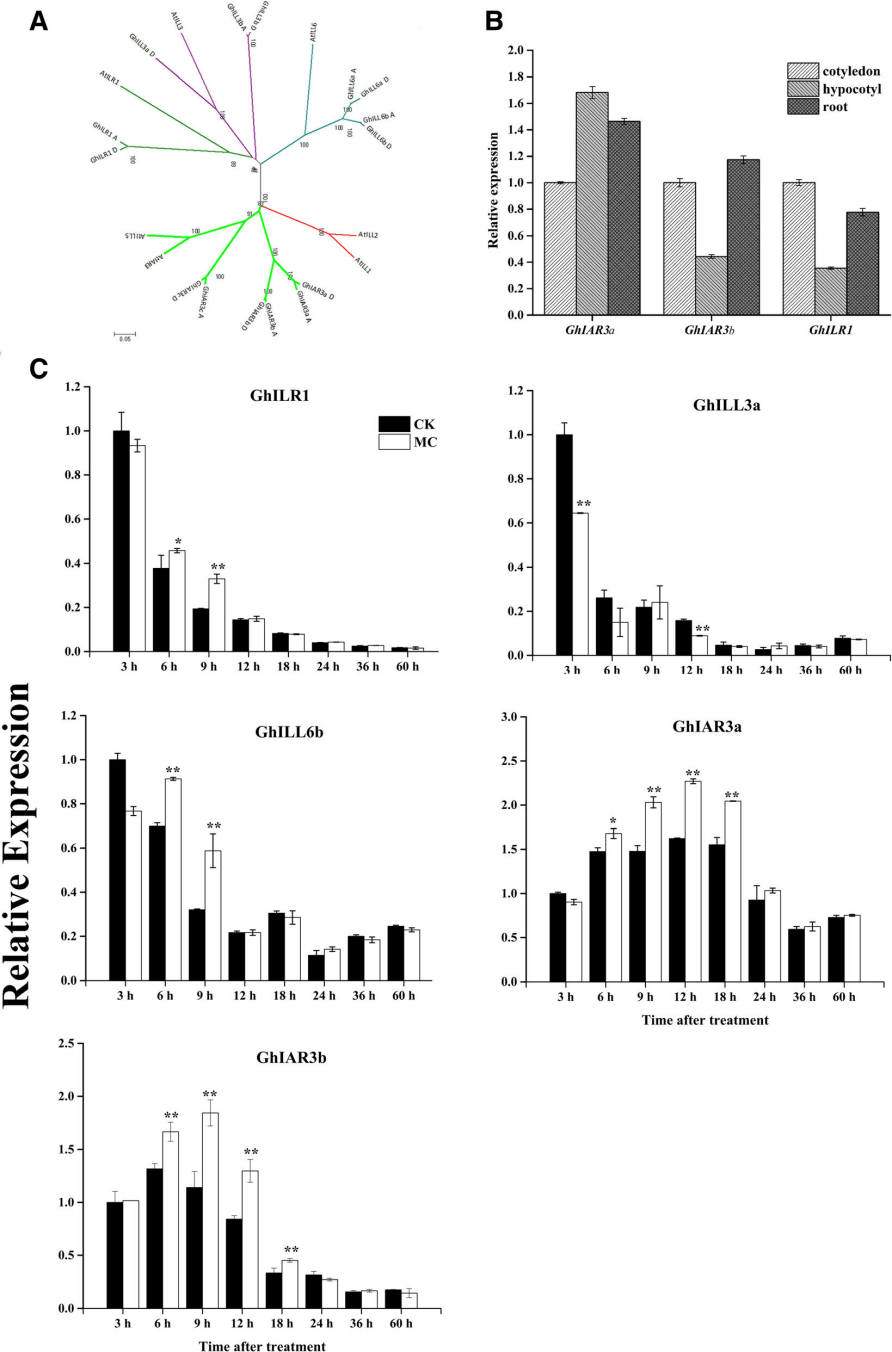
Analysis of IAA amidohydrolase family of cotton. **a** Phylogenetic tree of amidohydrolase homologues from Arabidopsis (*At*), cotton (*Gh*). A Neighbor-Joining tree was generated by MEGA 6 software with a bootstrap value of *n* = 1000, based on full-length protein alignment by ClustalW. The numbers on the tree branches indicate bootstrap probability values. The scale bar represents the number of amino acid substitutions per site. **b** Relative expression of *GhIAR3a, GhIAR3b* and *GhILR1* genes in various cotton tissues. **c** Expression of *GhILR1, GhILL3a, GhILL6b, GhIAR3a* and *GhIAR3b* (b) in control and MC-treated cotton seedlings. Values are the means ± SD (*n* = 4). Asterisks (*) and (**) indicate a significant difference (*P* < 0.05) and (*P* < 0.01), respectively, in comparison with the corresponding control

Sixteen proteins of the GhIAH family were found in *Gossypium hirsutum* (Additional file 2: Table S1). Phylogenetic analysis comparing the GhIAH family of proteins against all members of the AtIAH family confirmed that the majority (GhIAR3a, −b, and -c) are similar to AtIAR3. GhILR1, GhILL3a/b, and GhILL6a/b proteins are similar to AtILR1, AtILL3, and AtILL6 proteins, respectively (Fig. 5a).

The PCR results showed that *GhILR1*, *GhILL3a*, *GhILL6b*, *GhIAR3a* and *GhIAR3b* were expressed during seed germination in cotton (Additional file 1: Figure S3). The amino acids of the GhILR1, GhILL3a, and GhILL6b proteins showed 53, 58, and 62% identity with the AtILR1, AtILL3, and AtILL6 proteins, respectively (Additional file 1: Figure S5). GhIAR3a and GhIAR3b showed 63 and 64% identity with AtIAR3, respectively, and GhIAR3a and GhIAR3b were 83% identical (Additional file 1: Figure S4).

The relative expression of *GhILR1*, *GhILL6b*, *GhIAR3a* and *GhIAR3b* markedly increased in young cotton seedlings after MC treatment (Fig. 5c). The relative expression of *GhILR1* and *GhILL6b* increased 10–34.0% and 24.1–83.3%, respectively, at 6–9 h after treatment (Fig. 5c). The expression of *GhIAR3a* and *GhIAR3b* increased by 27.0–55.2% and 8.7–67.2%, respectively, in the cotton seedlings at 6–18 h after treatment (Fig. 5c). However, ILL6 enzymes exhibited no activity against IAA-amides in Arabidopsis [23, 24]. Thus, based on expression profiling, *GhILR1*, *GhIAR3a* and *GhIAR3b* were selected for functional validation. The spatial expression results showed that all those genes were expressed in the cotyledons, hypocotyls, and roots of cotton (Fig. 5b).

### Cloning and functional analysis of *GhIAH*s

*GhILR1*, *GhIAR3a* and *GhIAR3b* cDNAs were cloned. The predicted amino acids were aligned with the orthologs of *G. hirsutum* (*AD1*), *Gossypium raimondii* (D subgenome) and *Gossypium arboretum* (A subgenome), whose genomes have been sequenced (Additional file 1: Figures S5–7). The sequences showed the highest identity with the *G. hirsutum* D subgenome (Additional file 1: Figures S5–7). GhILR1, GhIAR3a and GhIAR3b comprised 428, 444 and 443 amino acids, respectively. GhIAR3a and GhIAR3b may be localized in the endoplasmic reticulum (ER) containing the RDEL/HEDL sequence that signals the retrieval of plant proteins to the ER lumen (Additional file 1: Figure S4). In addition, both have a cleavable N-terminal signal sequence, and according to the predicted results, the mature protein will start with codon 24 of GhIAR3a and GhIAR3b. GhILR1 also has a cleavable N-terminal signal sequence (the mature protein starts with codon 19) (Additional file 1: Figures S5–7).

*GhIAR3a* and *GhIAR3b* were expressed in *Escherichia coli* (BL21) as glutathione S-transferase (GST)-tagged fusion proteins, while GhILR1 was expressed in *E. coli* (BL21) as His-tagged fusion proteins. Purified GST-GhIAR3a, GST-GhIAR3b and His-ILR1 were tested for IAA conjugate hydrolysis via different IAA-amide conjugates (Additional file 1: Figure S8A). All three hydrolases cleaved several IAA-amide conjugates, including IAA-Gly, IAA-Leu, IAA-Met and IAA-Phe (Table 2). However, only GST-GhIAR3a efficiently hydrolyzed IAA-Ala (Table 2). The purified GST did not hydrolyze any of these conjugates.

**Table 2:** Substrate specificity of *Gossypium hirsutum* amidohydrolases

Conjugate	Hydrolase activity^a^ (μmol IAA released/min/mg)		
	His-GhILR1	GST-GhIAR3a	GST-GhIAR3b
IAA-Ala	-^b^	79±4.5	-
IAA-Asp	4.9±0.5	0.2±0.1	0.5±0.1
IAA-Gly	5.1±0.2	4±0.4	4.6±0.5
IAA-Ile	-	-	-
IAA-Leu	10±1.2	4.3±0.7	4.2±0.5
IAA-Met	6.3±1.0	8.2±0.9	3.8±0.3
IAA-Phe	12.3±1.6	5.1±0.8	5.4±0.7

^a^Values shown are the means of at least 3 time points ±S.D.s

^b^Not detected

### Preparation of mouse mAb and the development of a sandwich ELISA for measuring GhIAR3a

**Fig. 6:**
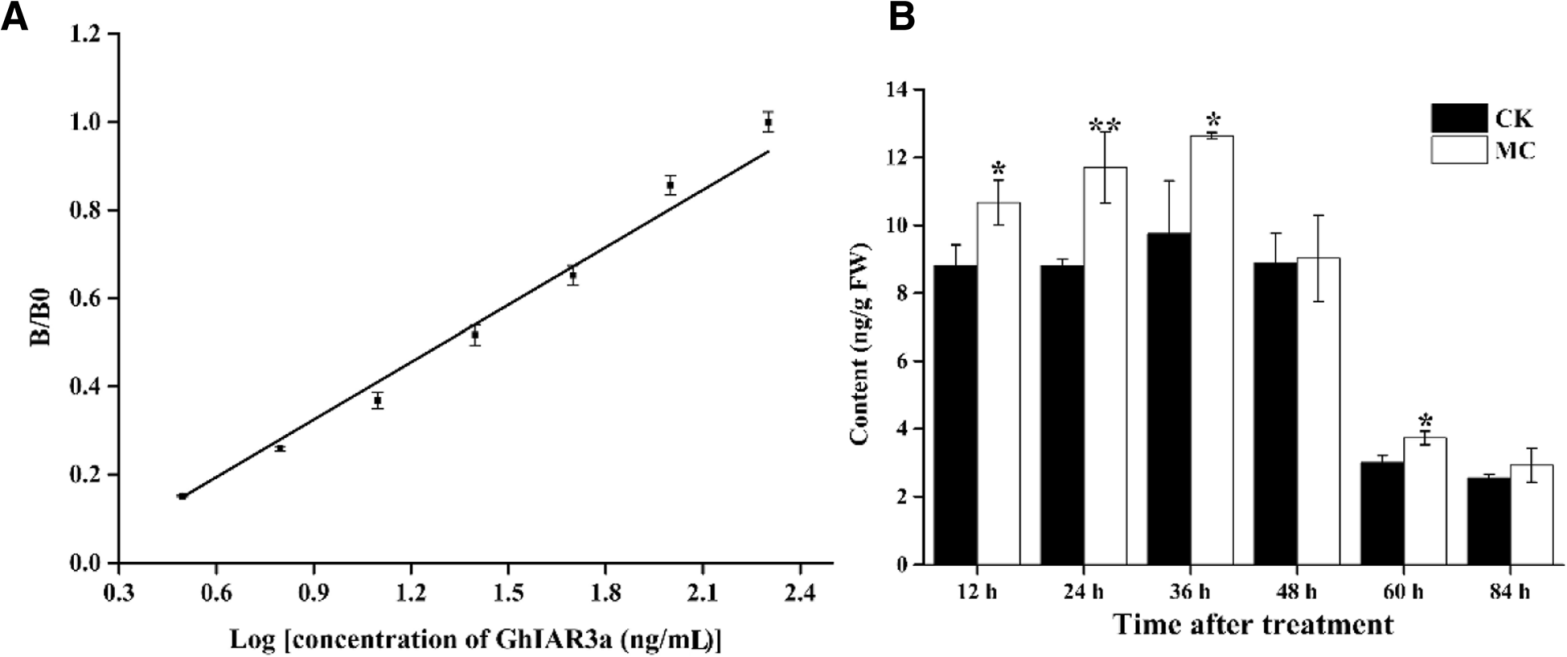
Standard curve for GhIAR3a ELISA under optimized conditions (**a**) and the content of GhIAR3a (**b**). B0 is absorbance at 492 nm in the highest concentration of GhIAR3a. B is absorbance at 492 nm in the various concentration of GhIAR3a. Asterisks (*) and (**) indicate a significant difference (*P* < 0.05) and (*P* < 0.01), respectively, in comparison with the corresponding control

GhIAR3a was separated from GST-GhIAR3a as the antigen (Additional file 1: Figure S8B), which was used to immunize rabbits and mice [30]. The mouse with the highest titer was used for cell fusion. The clone, designated 3D11, was selected by limiting dilution. The titer of rabbit antisera (the maximum serum dilution that yielded an absorbance of 1.0 under noncompetitive assay conditions) was 8–16 × 10^3^. The optimal concentrations of rabbit serum, mouse mAb, and peroxidase-labeled goat anti-mouse IgG were screened via checkerboard titration. A sandwich ELISA was established under optimal conditions (Fig. 6a). The working range for GhIAR3a was 0.5–200 ng/mL. The antibodies did not recognize GST, GhIAR3b or GhILR1 in the sandwich ELISA (Additional file 1: Figure S9). Although GhIAR3a and GhIAR3b showed 83% identity in amino acid sequences, the antibodies did not recognize GhIAR3b, possibly because the 3D structures of these two proteins were different. In in vitro experiments, GhIAR3a cleaved IAA-Ala efficiently, while GhIAR3b could not hydrolyze IAA-Ala.

The GhIAR3a content in cotton seedlings was measured via an established sandwich ELISA. The content markedly increased by 41.7~84.3% in MC-treated seedlings at 12–60 h (Fig. 6b), which was consistent with the upregulated expression of *GhIAR3a* in response to MC treatment.

### The Arabidopsis *iar3* mutant exhibited a reduced response to MC treatment

**Fig. 7:**
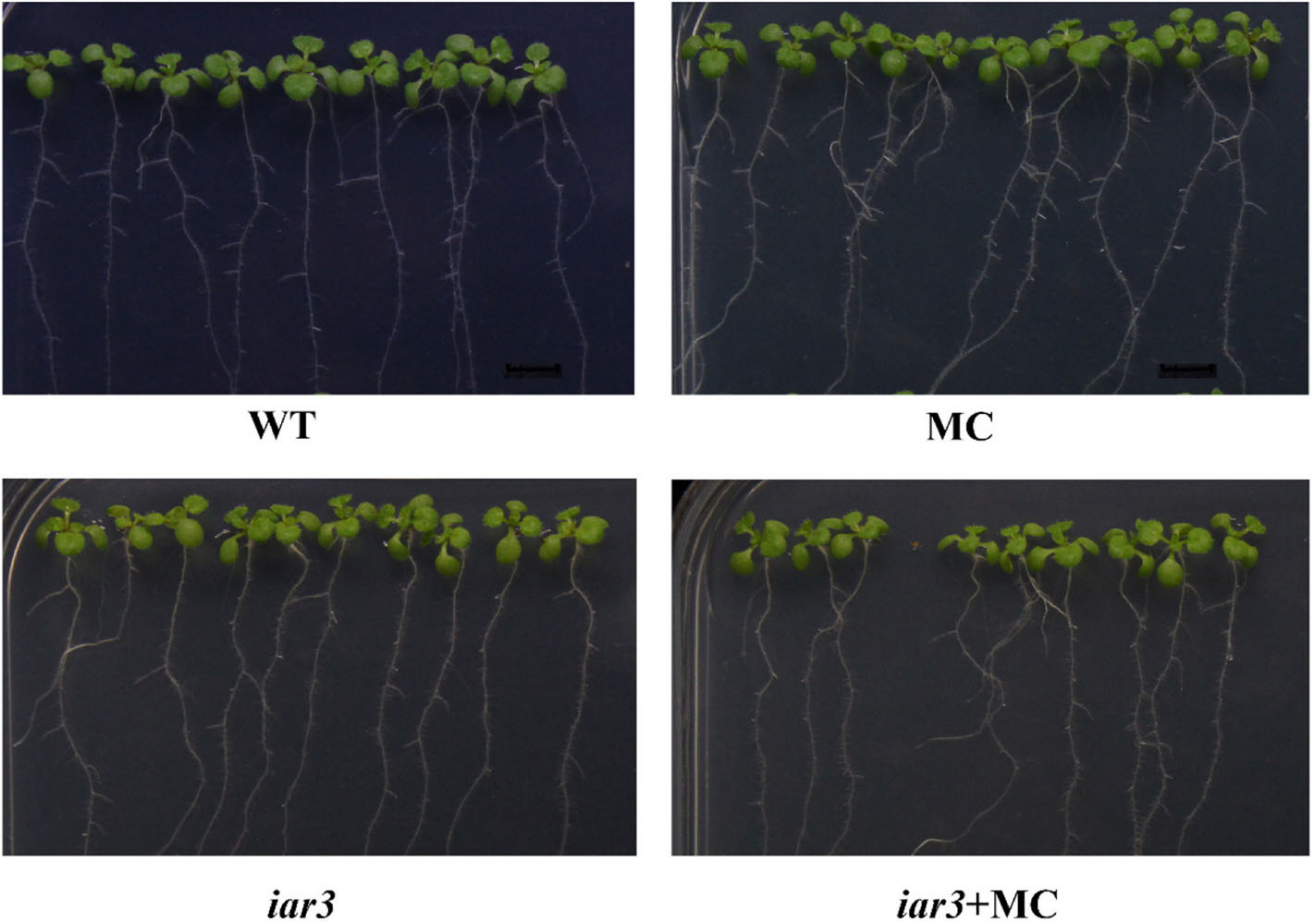
Root phenotypes of Arabidopsis wild-type and mutant (*iar3*) under 100 μM MC treatment in culture media

To identify whether *IAR3* genes are responsible for the effects of MC on LR formation during the seedling stage, the phenotypes of the Arabidopsis *iar3* mutant and Columbia-0 (Col-0) wild-type plants under 100 μM MC treatment were studied. Arabidopsis *iar3* mutants have a T-DNA insertion in the *AtIAR3* (AT1G51760) promoter. The amino acid sequences of GhIAR3a and GhIAR3b are most similar to the amino acid sequence of the AtIAR3 protein. Enzymatic analysis of GhIAR3a revealed that GhIAR3a preferentially hydrolyzes IAA-Ala (Table 2), which is in accordance with results concerning Arabidopsis AtIAR3 (Sherry et al., 2002). The number of LRs markedly increased by 38.6% at 10 dat in the Col-0 wild-type seedlings but increased by only 7.8% in the *iar3* mutant seedlings (Fig. 7). These results demonstrate that *GhIAR3* might play an important role in LR formation in cotton seedlings in response to MC treatment.

## Discussion

MC application to cotton seeds can increase root growth by increasing root length and the number of LRs [4, 6]. These phenomena were confirmed by our data, which showed that MC treatment induced an increase in LR number and elongation. Several studies have reported that the determination of the sites of LRs occurs in advance of the visible initiation of LRP. Moreover, the division of LRI occurred earlier in MC-treated seedlings than in control seedlings, suggesting that the increased LR number was largely the result of increased LRI in MC-treated seedlings.

It is well known that LRs originate from pericycle cells. The priming of pericycle cells for LRI occurs in the basal meristem between the apical meristem and the first division region of LRI [29] and has the potential to initiate a LR [11]. The first division leading to the formation of LRP occurs 3 mm behind the root tip in Arabidopsis [31]. However, the first divisions occurred approximately 10–13 mm behind the root tip in radish and approximately 12–15 mm behind the root tip in sunflower, corn, and carrot roots [32]. We found that the first divisions occurred approximately 15–20 mm behind the root tip in cotton (Fig. 3a).

IAA plays a central role in regulating the priming and divisions of pericycle cells during LRI [11]. The IAA transcription factors ARF7 and ARF19 are then activated and modulate the transcription of their target genes, such as *LBD16* and *LBD18* [14, 33]. MC significantly increased the IAA level in the priming region of cotton roots and consistently upregulated the expression of *GhARF7*, *GhARF19*, *GhLBD18–1* and *GhLBD18–2* in the roots of cotton seedlings treated with MC (Fig. 4).

Hydrolysis of IAA conjugates represents the principal source of IAA in both germinating seeds and developing seedlings [18, 19]. In this study, an evident rapid hydrolysis of IAA-amide conjugates was observed during cotton seed germination, showing a marked reduction in the levels of IAA-Phe, IAA-Met, IAA-Ala conjugates (Table 1). Compared with that in the control seedlings, the content of IAA-amide conjugates in the MC-treated seedlings was reduced (Table 1). This finding indicated that the increase in IAA content in response to the MC treatment might be carried out by accelerating the hydrolysis of IAA-amide conjugates.

IAH genes have been identified in many plant species, including gymnosperms, monocots, and dicots [34]. To date, seven genes have been identified in the *AtIAH* family: *ILR1*, *ILL1*, *ILL2* and *IAR3*, which encode enzymes that can cleave IAA-amide conjugates to some extent in vitro [22–24]; *ILL3* and *ILL6/GR1*, which have no such catalytic activity; and *ILL5*, which is supposedly a pseudogene [23]. Database searches confirmed that sixteen *GhIAH*s were assumed in *G. hirsutum* and show high homology to *AtIAH*s*.* Our results showed that the relative expression of *GhILL6*, *GhILR1*, *GhIAR3a* and *GhIAR3b* was upregulated in the MC-treated seedlings (Fig. 5c). *MtIAR33* and *MtIAR34* gene expression was dramatically upregulated after inoculation with *Sinorhizobium meliloti* and *Glomus intraradices* [28]. Additionally, their hydrolase activity increased, and the expression of the *Br-IAR3 25* and *Br-ILL6* genes was altered in infected root galls in Chinese cabbage [27, 35].

As described for *IAR3* in Arabidopsis, *B. rapa* and *M. truncatula* [23, 27, 28], both *GhIAR3a* and *GhIAR3b* have in their amino acid sequence a possibly cleavable N-terminal signal sequence, and both terminate with the HDEL/RDEL, which signals retention to the lumen of the ER. Similar to Arabidopsis *ILR1*, *GhILR1* also has a cleavable N-terminal signal sequence [22]. To test in vitro activity, we generated recombinant proteins of GhIAR3a and GhIAR3b with an N-terminal GST tag and GhILR1 with a C-terminal 6× His tag. All these hydrolases cleaved several IAA-amide conjugates. IAA-Ala was the best substrate of GhIAR3a, while GhILR1 hydrolyzed IAA-Phe and IAA-Leu most efficiently. GhILR1 also hydrolyzed IAA-Asp, IAA-Gly and IAA-Met to some extent in vitro. These findings are in accordance with results for Arabidopsis IAR3 and ILR1 [24]. GhIAR3a and GhIAR3b cleaved IAA-Gly, IAA-Met, IAA-Leu and IAA-Phe at similar rates, whereas GhIAR3b could not hydrolyze IAA-Ala (Table 2). These results indicated that IAA-Ala was cleaved only by GhIAR3a.

Rabbit polyclonal antibodies and mouse mAbs against GhIAR3a were developed. Based on these antibodies, a double sandwich ELISA system was established to detect GhIAR3a. Our results showed that the content of GhIAR3a increased in MC-treated seedlings compared with the control seedlings (Fig. 6b). IAR3 is highly conserved and may plays major roles in physiological function [36]. We identified the majority of IAR3 orthologs in cotton (Fig. 5a). In addition, compared with wild-type Arabidopsis, the mutant *iar3*, which is defective in amidohydrolase via a T-DNA insertion within the *AtIAR3* gene, exhibited a reduced response to MC-induced LR development (Fig. 7).

## Conclusions

The application of MC increased the endogenous IAA content, leading to the promotion of LRI by upregulating *GhARF7/19* and *GhLBD18*s and subsequently increasing the LR number and elongation. MC upregulated the expression of *GhIAR3a*, *GhIAR3b* and *GhILR1*, which encode amidohydrolases that could cleave IAA-Phe, IAA-Met, IAA-Gly, IAA-Leu and IAA-Ala to free IAA in vitro, accelerating the hydrolysis of IAA-amide conjugates to generate more IAA in cotton seedlings. These results suggested that the application of MC could mediate IAA and IAA conjugate homeostasis to promote LR development. Moreover, compared with that in the control seedlings, the GhIAR3a content in the MC-treated seedlings also increased, as revealed via an established sandwich ELISA. In addition, the Arabidopsis *iar3* mutant exhibited a reduced response to MC-induced LR development. These findings provide novel insights into the molecular understanding of increases in LR number in response to MC. Additional research is needed to investigate how MC regulates IAH genes.

## Methods

### Hydroponic culture of cotton and treatment methods

Cotton variety Xinkang 4 was used. The MC standard (97.0% purity) was supplied by Hebei Guoxin ahadzi-nonon Biological Technology Co., Ltd. (Heibei, China). The MC treatment involved soaking seeds with the MC solution at 30 °C for 12 h.

After they were soaked, the seeds were incubated for three days in rolls of germination paper (25 cm wide, 38 cm long, catalog number OP1015, Hoffman Manufacturing, Inc., Albany, USA) at 25 °C and 70% relative humidity. Eight cotton seedlings were then cultured via hydroponics with Hoagland nutrient solution in a pot under a 14 h photoperiod, a 24/20 °C day/night temperature cycle, a 550 μmol/m^2^/s light intensity, and a 60% relative humidity. Four seedlings were treated with MC, while the other four were MC free.

The number of LRs was measured at 6, 12 and 20 d after MC treatment. Biomass was measured at 12 and 20 d after treatment.

### Arabidopsis Murashige and Skoog (MS) culture medium

Arabidopsis Col-0 and *iar3* mutant (SALK_042101C) plants were grown in MS media. The seeds were surface sterilized and grown on plant nutrient media containing 3% (*w*/*v*) sucrose solidified with 0.8% agar. One hundred micromolar MC was diluted into each medium from 100 mM stocks in water. The growth conditions were as follows: a constant 16 h photoperiod with a temperature cycle of 22/20 °C day/night, a light intensity of 25–45 μmol/m^2^/s, and a relative humidity of 60%. The LRs were imaged and counted under a dissecting microscope Olympus SEX16 (Tokyo, Japan) at 10 d after MC treatment.

### Inspection of LRP

Each primary root was fixed in formaldehyde-acetic acid-alcohol (FAA) (5% formaldehyde, 5% acetic acid, and 50% ethanol) for 12–24 h and then stored in 70% ethanol. The LRP were dyed using Schiff’s reagent, hyalinized by gradient glycerol, and inspected under a stereoscope Olympus SEX16 (Tokyo, Japan)without slicing the roots [37].

### Root scanning and image processing of LRs

The roots were scanned directly by an Epson scanner, and the images were analyzed using RHIZO 2004b software. The topological connections and geometric data of individual roots were analyzed using a Visual Basic for Applications program [38].

### Microscopy analysis of LR initiation

Twenty-five millimeters of the most apical region of the roots was dissected and divided into 5 mm long segments at 2 d after MC treatment. The region that was 5–10 mm behind the root base was dissected at 2 and 3 d after treatment. The roots were fixed in FAA solution under vacuum and then embedded in paraffin. Sections of the roots were cut into serial longitudinal sections that were 8 μm thick. The samples on slides were deparaffinized and stained with safranin-fast green. Microscopy was conducted with an Olympus DP 73 microscope (Tokyo, Japan).

### Quantitative analysis of endogenous IAA and IAA-amide conjugates

IAA, IAA-Ala, IAA-Ile, IAA-Phe and ^2^H-labeled IAA were purchased from Sigma-Aldrich (Sigma-Aldrich, USA). The remaining conjugates (IAA-Leu, IAA-Met and IAA-Gly) were synthesized according to the methods of LeClere (2002) [24]. The plant hormones in the crude plant extracts were quantitatively analyzed via high-performance liquid chromatography-electrospray ionization-tandem mass spectrometry (HPLC-ESI-MS/MS) [39]. Briefly, frozen plant tissues were ground in liquid nitrogen and weighed, after which each sample (50 mg) was transferred to a 1.5 mL screw-cap tube. Fifty nanograms of the working solution of ^2^H-labeled IAA was then added to each tube. A total of 500 μL of extraction solvent, 2-propanol/H_2_O/concentrated HCl (2:1:0.002, *v*/v/v), was added to each tube. The tubes were shaken under 100 rpm for 30 min at 4 °C. Afterward, 1 mL of dichloromethane was added to each sample, after which the samples were shaken for 30 min at 4 °C and then centrifuged at 13,000 g for 5 min. The filtered extracts were evaporated using a nitrogen (N_2_) evaporator. The samples were redissolved in 0.1 mL of methanol, and 50 μL of sample solution was injected into a reverse-phase C18 Gemini HPLC column for HPLC-ESI-MS/MS analysis. The data were then analyzed using the software Xcalibur 2.0 (Thermo-Finnigan, California, USA).

### Immunohistochemical localization of IAA

Twenty roots (the area 0.3–1.5 cm behind the root tip) were sampled randomly and divided into 4 mm long segments. The samples were immediately prefixed in a 2% (*w*/*v*) aqueous solution of 1-ethyl-3-(3-dimethylaminopropyl)-carbodiimide (EDC, Sigma--Aldrich, USA) for 1 h, postfixed overnight at 4 °C in a solution containing 4% paraformaldehyde and 2.5% glutaraldehyde, dehydrated with a graded ethanol series, embedded in paraffin, and ultimately sectioned into 8 μm slices. The slides were spread with polylysine before the sections were fixed.

The dried sections were deparaffinized with xylene and hydrated in an ethanol-water series. The slides were processed as described in [40], with some modifications. Primary IAA antibodies were developed in our laboratory. A drop of 100 μL of primary IAA antibodies (10 μg/mL in 10 mM phosphate-buffered saline (PBS, pH 7.0, containing 0.2 g/L KCl, 2.19 g/L Na_2_HPO_4_. 12H_2_O, 0.482 g/L KH_2_PO_4_)) was added to each slide before the inner membrane was covered with plastic gloves and then incubated overnight in a humidity chamber at 4 °C. One hundred microliters of secondary antibodies [5 μg/mL anti-mouse IgG-FITG in PBS, Promega, USA] was then added to each slide, which was then incubated at 37 °C for 2 h. Microscopy was conducted with a Zeiss LSM710 microscope (Carl Zeiss, Germany).

### Analysis of the IAH family from *G. hirsutum*

To identify cotton IAH genes (*GhIAH*s), the full-length protein sequences of Arabidopsis AtIAHs were used as queries for a BLASTp analysis against the *G. hirsutum* NCBI protein database (http://www.cottongen.org/tools/blast). Amino acid alignments of members of the GhIAH family and Arabidopsis orthologs were performed using MEGA 6 software. Considering that tetraploid *G. hirsutum* has two subgenomes, lowercase and uppercase letters were used to distinguish closely related members. An uppercase ‘A’ was further added to indicate that the gene is from the A subgenome or an uppercase ‘D’ for the D subgenome. If multiple members are co-orthologous to the closest AtIAH, lowercase letters (a-c) were added after the numbers to differentiate genes within the same subclade. The PCR primers were constructed to recognize homologous gene pairs (*Gh_A* and *Gh_D)* because of sequence conservation.

### RNA extraction and quantitative real-time PCR (qRT-PCR) analysis

Total RNA was extracted with a Plant RNeasy Mini kit (Qiagen, Germany) according to the manufacturer’s instructions. Two micrograms of total RNA was treated with DNase I and used for cDNA synthesis with oligo (dT) primers and reverse transcriptase (Promega, USA). qRT-PCR was carried out on an Applied Biosystems 7500 Fast Real-Time PCR System (Applied Biosystems, USA); the reaction volume was 15 μL and comprised 1.5 μL of diluted cDNA, 0.3 μL of ROX reference dye, 0.3 μL of each forward primer and reverse primer (each at 10 μM), and 7.5 μL of SYBR Premier Ex Taq mix (Takara, Japan). PCR amplification was performed using two-step cycling conditions of 95 °C for 30 s, followed by 40 cycles of 95 °C for 5 s and 60 °C for 35 s. The primers used are listed in Additional file 1: Table S2. The levels of each gene transcript were calculated relative to those in corresponding untreated controls. Fold changes of RNA transcripts were calculated by the 2^-∆∆Ct^ method [41], and the values reported represent the average of three independent trials.

### Generation and purification of fusion proteins

*GhIAR3a* and *GhIAR3b* (without the predicted N-terminal signal sequences) were expressed in *E. coli* as fusions to the N terminus of a GST tag, while *GhILR1* was fused to the N terminus of a His tag. *pGEX-IAR3a* and *pGEX-IAR3b* recombinant genes were generated by introducing an *EcoR*I site at codons 23–24 in the cDNA and subcloning the *EcoR*I-*Sal*I fragment into pGEX-4 T-1 that was cut with the same restriction enzymes. The recombinant gene *pET30-ILR1* was subcloned into a pET30a-(c) vector.

With respect to cytoplasmic protein expression, the constructs were transformed into *E. coli* strain BL21 (DE3) via the heat shock method. A single colony of transformed cells was inoculated in 5 mL of LB^+^ (1% tryptone, 0.5% yeast extract, 0.5% NaCl, 0.2% glucose containing 100 μg/mL ampicillin for *pGEX-IAR3a* and *pGEX-IAR3b* or 50 μg/mL kanamycin for *pET30-ILR1*) at 30 °C under shaking at 200 rpm overnight. Afterward, 1.2 mL of the overnight culture was transferred into 120 mL of LB^+^ and shaken at 30 °C for 7~8 h. The cultures were induced by adding 0.3 mM isopropyl β-D-thiogalactoside under shaking at 20 °C overnight. The proteins were purified as described previously [23] and stored at − 80 °C until analysis. The proteins were later quantified spectrophotometrically.

### Enzyme assays and determination of hydrolysis rates

Preliminary hydrolytic activity was measured as described previously [24]. Briefly, the reactions comprised 50 mM Tris-HCl (pH 8.0), 1 mM dithiothreitol (DTT), 1 mM MnCl_2_, 1 mM IAA-amino acids, and a GST-IAR3a or GST-IAR3b fusion protein within a range of concentrations from 7.5 to 60 ng/μL. Reactions contained 50 mM Tris-HCl, pH 7.5, 1 mM DTT, 1 mM MnCl_2_, 1 mM IAA-amino acid, and the fusion protein of His-ILR1 within a range of concentrations from 7.5 to 60 ng/μL. The reactions were incubated at room temperature for 16–24 h. At each time point, 100 μL of the reaction was stopped by dilution in 900 μL of methanol and 1% acetic acid and stored at − 20 °C until HPLC analysis.

### HPLC analysis

The percentage of IAA released from each conjugate was determined via HPLC [42]. Following centrifugation to insoluble pellet material, 10 μL of each sample was injected onto a 4.6 mm reversed-phase C18 column (5 μM× 250 mm, Thermo Fisher Scientific, USA) whose mobile phase consisted of methanol and 1% acetic acid (60:40 *v*/v) in isocratic mode at a flow rate of 1 mL/min. The detection was monitored at 282 nm.

### Preparation of GhIAR3a antibodies

Monoclonal anti-GhIAR3a antibodies were generated in accordance with previously described procedures [43]. Briefly, Balb/c mice were immunized with GhIAR3a protein. Hybridomas were obtained by fusing the spleen cells of the best performing mouse and Sp2/0 mouse myeloma cells. The hybridomas were selectively cultured, and the supernatants were screened by an ELISA. The resulting mAbs were generated by inoculating the selected hybridoma cells into Balb/c mice. GhIAR3a mAbs were purified from ascitic fluids by ammonium sulfate precipitation.

Rabbit polyclonal antibodies were generated by immune back and thigh muscles of 3-month-old male New Zealand rabbits and subsequently injected two more times at 2-week intervals [30]. Rabbit serum was subsequently obtained from rabbit heart tissue.

### Sandwich ELISA procedure

Sandwich ELISAs was performed as follows: Wells of microtiter plates were coated with rabbit serum and diluted in 0.05 M carbonate buffer at 37 °C for 3 h. Fifty microliters of standard-sample GhIAR3a or plant extract was then added to each well, followed by 50 μL of mAbs at 37 °C for 30 min. The subsequent procedure was performed as described in [43]. The data were calculated via OriginPro 9 (OriginLab) software.

### Statistical analyses

All statistical analyses were performed using the Univariate General Linear Models procedure of SPSS 20. Least significant differences (LSDs) were used to separate treatment means at *P* < 0.05 and *P* < 0.01.

## Additional files

Additional file 1: **Figure S1.** Changes in root, shoot biomass and shoot/root ratio at 12 (A) and 20 (B) d after MC treatment. **Figure S2.** The effect of MC on IAA distribution in LRI priming region of cotton roots at 3 d after MC treatment. **Figure S3.** The expression of IAA amidohydrolase genes in the cotton seedlings. **Figure S4.** Amino acid sequence alignment of IAHs in cotton (*Gh*) and Arabidopsis (*At*). **Figure S5.** Amino acid sequence alignment of ILR1 in *Gossypium hirsutum* (CGP-BGI Assembly: CotAD_; NAU-NBI Assembly: Gh_A/Gh_D; Ncbi: XP_.) and *Gossypium raimondii* (JGI assembly: Gorai.). **Figure S6.** Amino acid sequence alignment of IAR3a in *Gossypium hirsutum*, *Gossypium arboretum* (BGI Assembly: Cotton_A) and *Gossypium raimondii* (BGI-CGP v1.0 Assembly: Cotton_D). **Figure S7.** Amino acid sequence alignment of IAR3b in *Gossypium hirsutum*, *Gossypium arboretum* and *Gossypium raimondii*. **Figure S8.** Purified fusion protein GST-IAR3a, GST- IAR3b and His-ILR1 used in activity assays (A) and GhIAR3a was separated as the antigen (B). **Figure S9.** Cross-Reactivity of mAb 3D11 and rabbit antisera against to GhIAR3a with GST (A), GhILR1 (B) and GhIAR3b (C). (DOCX 2404 kb)

Additional file 2: **Table S1.** Gene IDs and corresponding names of the cotton *GhIAH* family. **Table S2.** Primers. (XLSX 12 kb)

## Abbreviations

Ala: Alanine
ARF: Auxin response factor
Asp: Aspartic acid
Col-0: Columbia-0
dat: Days after MC treatment
DTT: Dithiothreitol
ELISA: Enzyme-linked immunosorbent assay
ER: Endoplasmic reticulum
FAA: Formaldehyde-acetic acid-alcohol
FW: Fresh weight
GA: Gibberellic acid
Glu: Glutamic acid
Gly: Glycine
HPLC-ESI-MS/MS: High-performance liquid chromatography-electrospray ionization-tandem mass spectrometry
IAA: Indole-3-acetic acid
IAH: IAA amidohydrolase
Ile: Isoleucine
LBD/ASL: Lateral organ boundaries-domain/asymmetric leaves2-like
Leu: Leucine
LR: Lateral root
LRI: Lateral root initiation
LRP: Lateral root primordia
mAb: Monoclonal antibody
MC: Mepiquat chloride
Met: Methionine
PBS: Phosphate-buffered saline
Phe: Phenylalanine
qRT-PCR: Quantitative real-time PCR
