# Supplementary material for: The effects of mepiquat chloride on the lateral root initiation of cotton seedlings are associated with auxin and auxin-conjugate homeostasis
Source: BMC Plant Biol. 2018 Dec 18;18:361. doi: 10.1186/s12870-018-1599-4 (PMC6299555; doi:10.1186/s12870-018-1599-4)
Supplement: Supplementary file 1 — Figure S1. Changes in root, shoot biomass and shoot/root ratio at 12 (A) and 20 (B) d after MC treatment. Figure S2. The effect of MC on IAA distribution in LRI priming region of cotton roots at 3 d after MC treatment. Figure S3. The expression of IAA amidohydrolase genes in the cotton seedlings. Figure S4. Amino acid sequence alignment of IAHs in cotton (Gh) and Arabidopsis (At). Figure S5. Amino acid sequence alignment of ILR1 in Gossypium hirsutum (CGP-BGI Assembly: CotAD_; NAU-NBI Assembly: Gh_A/Gh_D; Ncbi: XP_.) and Gossypium raimondii (JGI assembly: Gorai.). Figure S6. Amino acid sequence alignment of IAR3a in Gossypium hirsutum, Gossypium arboretum (BGI Assembly: Cotton_A) and Gossypium raimondii (BGI-CGP v1.0 Assembly: Cotton_D). Figure S7. Amino acid sequence alignment of IAR3b in Gossypium hirsutum, Gossypium arboretum and Gossypium raimondii. Figure S8. Purified fusion protein GST-IAR3a, GST- IAR3b and His-ILR1 used in activity assays (A) and GhIAR3a was separated as the antigen (B). Figure S9. Cross-Reactivity of mAb 3D11 and rabbit antisera against to GhIAR3a with GST (A), GhILR1 (B) and GhIAR3b (C). (DOCX 2404 kb) [file 12870_2018_1599_MOESM1_ESM.docx]

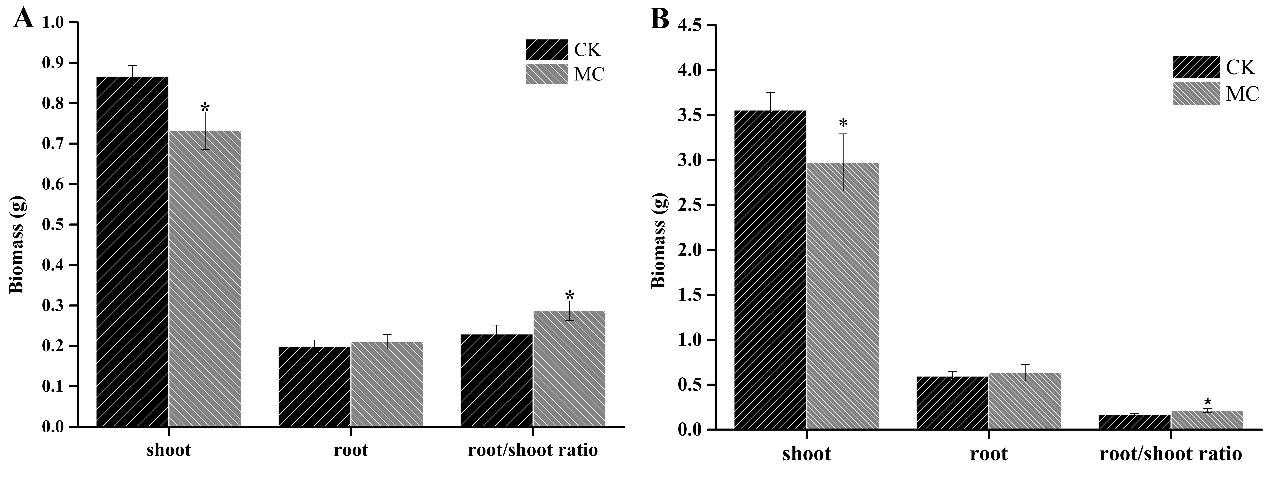


Figure S1. Changes in root, shoot biomass and shoot/root ratio at 12 (A) and 20 (B) d after MC treatment.

Values are the means ± SD (n = 3). Asterisks (*) indicate a significant difference (P < 0.05) compared with the corresponding control.


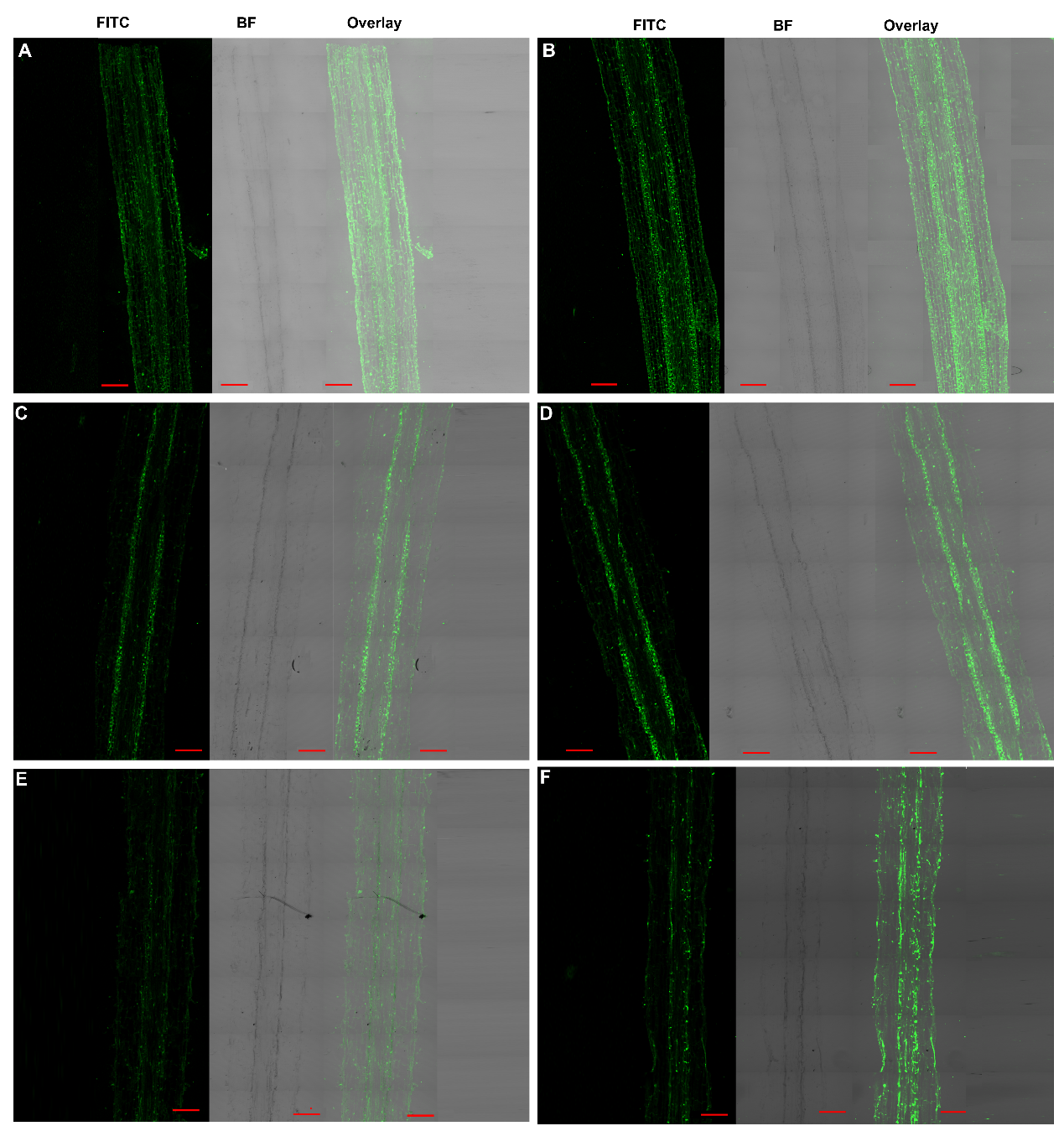


Figure S2. The effect of MC on IAA distribution in LRI priming region of cotton root at 3 d after MC treatment.

(A) to (F) Immunolocalization of IAA from the region of 0.3-0.7, 0.7-1.1, 1.1-1.5 cm behind the root tip in the control (A, C, E) and MC-treated (B, D, F) cotton seedlings.

Fluorescent images are labeled FITC, bright field (BF) and the corresponding overlay images (overlay). Bar = 200 µm.


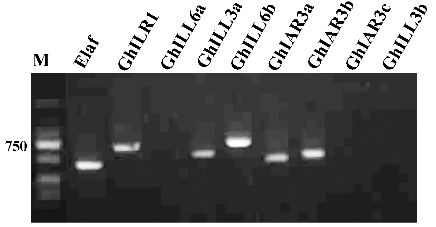


Figure S3.The expression of IAA amidohydrolase genes in the cotton seedlings.


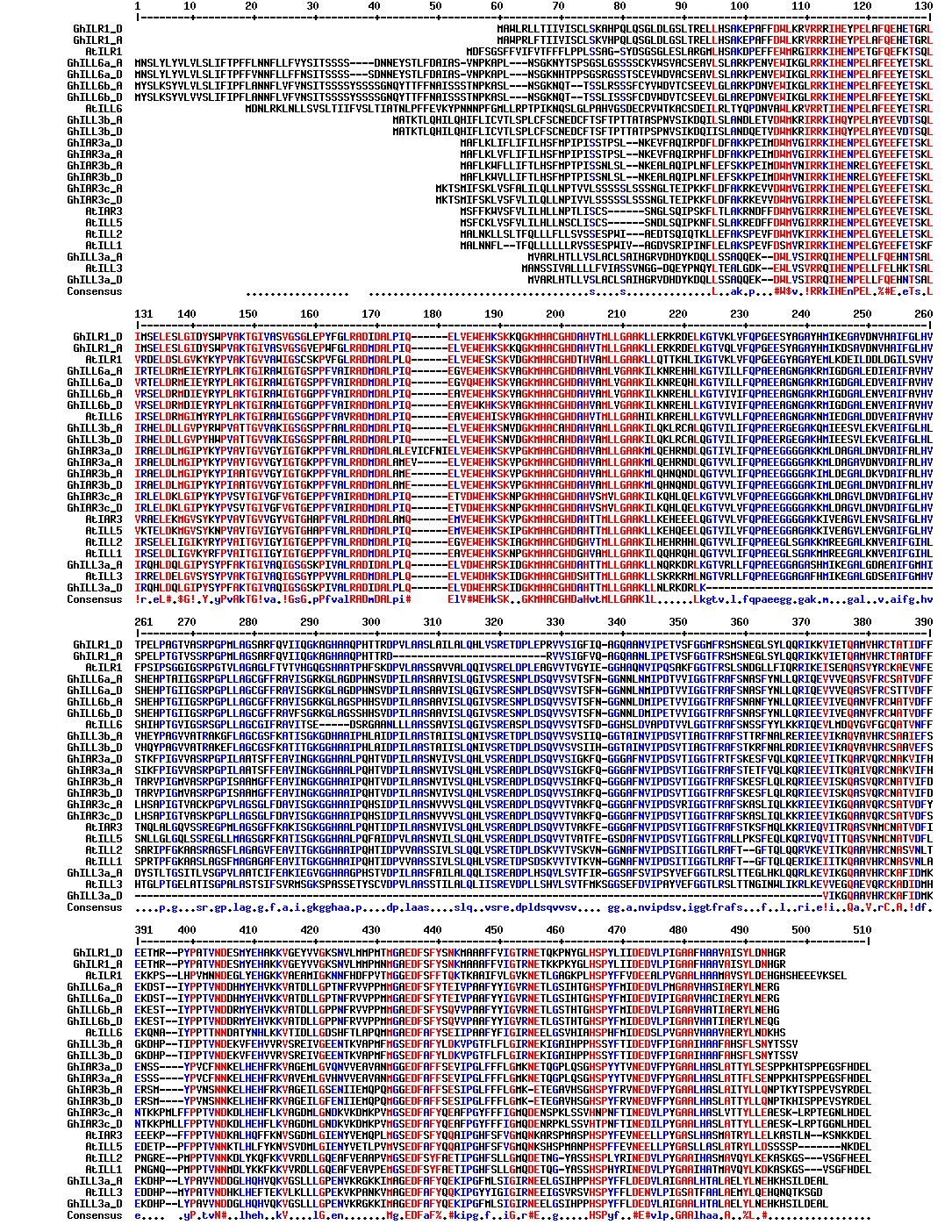


Figure S4. Amino acid sequence alignment of IAHs in cotton (*Gh*) and Arabidopsis (*At*). Alignment was performed using the Multalin website (http://multalin.toulouse.inra.fr/multalin/) with a hierarchical clustering approach.

*
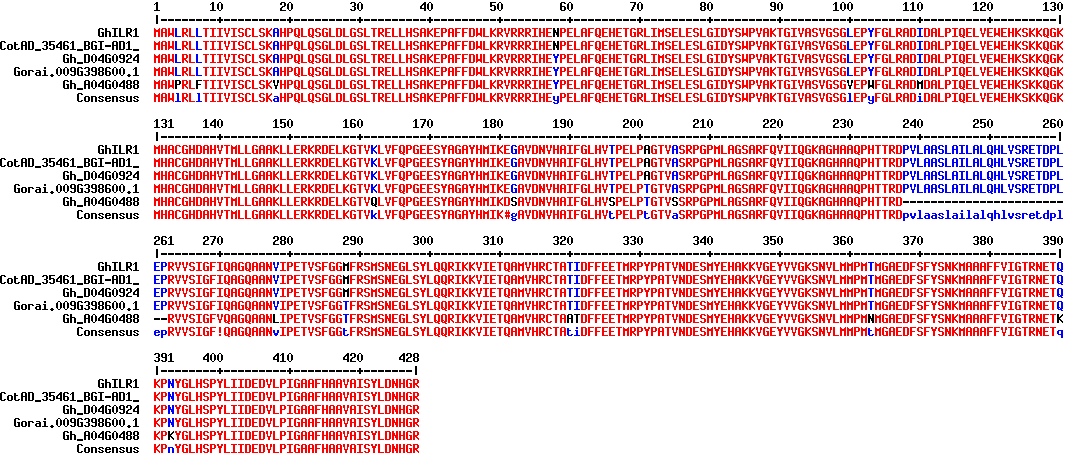
*

Figure S5. Amino acid sequences alignment of ILR1 in *Gossypium hirsutum* (CGP-BGI Assembly: CotAD_; NAU-NBI Assembly: Gh_A/Gh_D; Ncbi: XP_.) and *Gossypium* *raimondii* (JGI assembly: Gorai. ). Alignment was performed using the Multalin website (http://multalin.toulouse.inra.fr/multalin/) with a hierarchical clustering approach. The green box indicated N-terminal signal sequence.


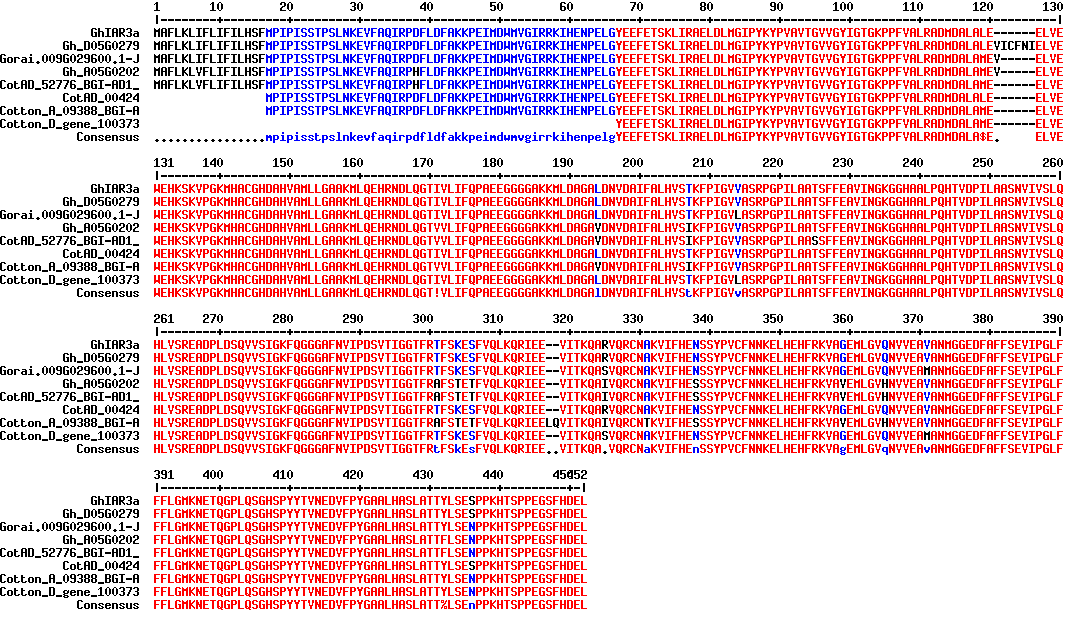


Figure S6. Amino acid sequences alignment of IAR3a in *Gossypium hirsutum*, *Gossypium arboretum* (BGI Assembly: Cotton_A) and *Gossypium* *raimondii* (BGI-CGP v1.0 Assembly: Cotton_D). Alignment was performed using the Multalin website (http://multalin.toulouse.inra.fr/multalin/) with a hierarchical clustering approach. The green box indicated N-terminal signal sequence.


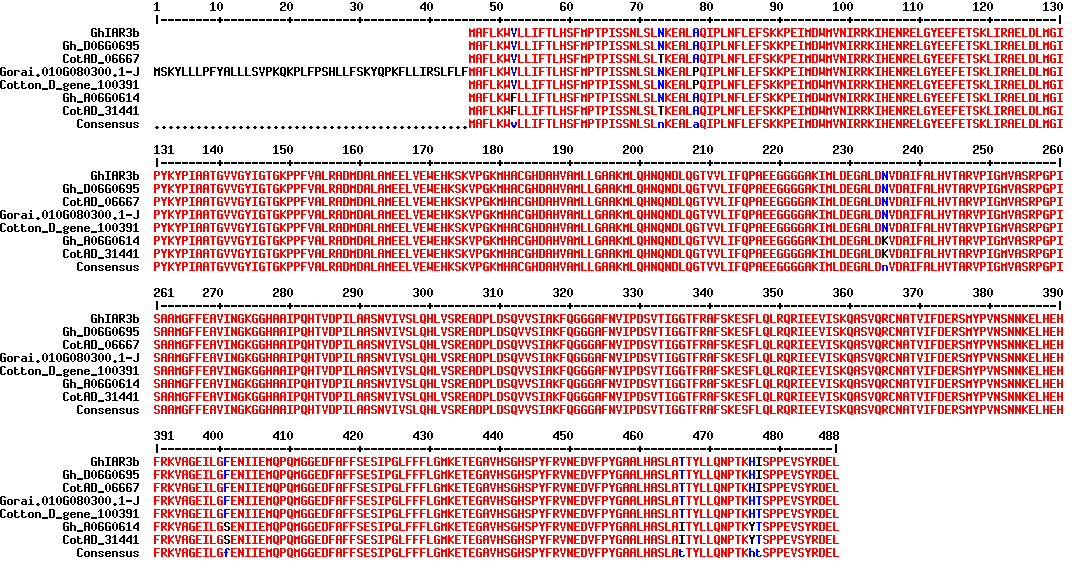


Figure S7. Amino acid sequences alignment of IAR3b in *Gossypium hirsutum*, *Gossypium arboretum* and *Gossypium* *raimondii*. Alignment was performed using the Multalin website (http://multalin.toulouse.inra.fr/multalin/) with a hierarchical clustering approach. The green box indicated N-terminal signal sequence.


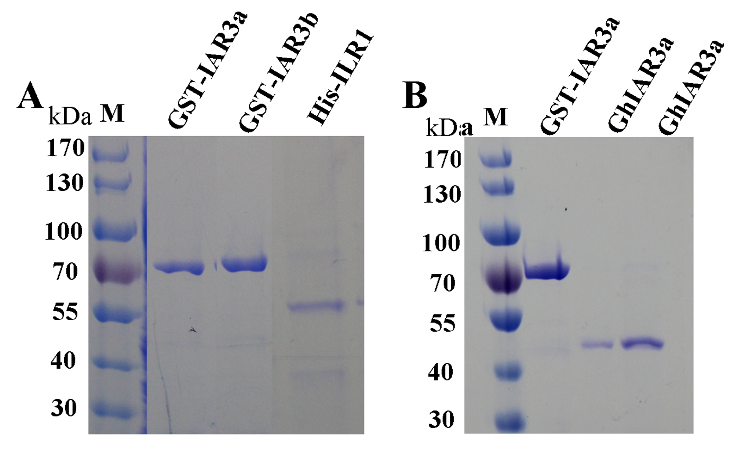


Figure S8. Purified fusion protein GST-IAR3a, GST- IAR3b and His-ILR1 used in activity assays (A) and GhIAR3a was separated as the antigen (B).


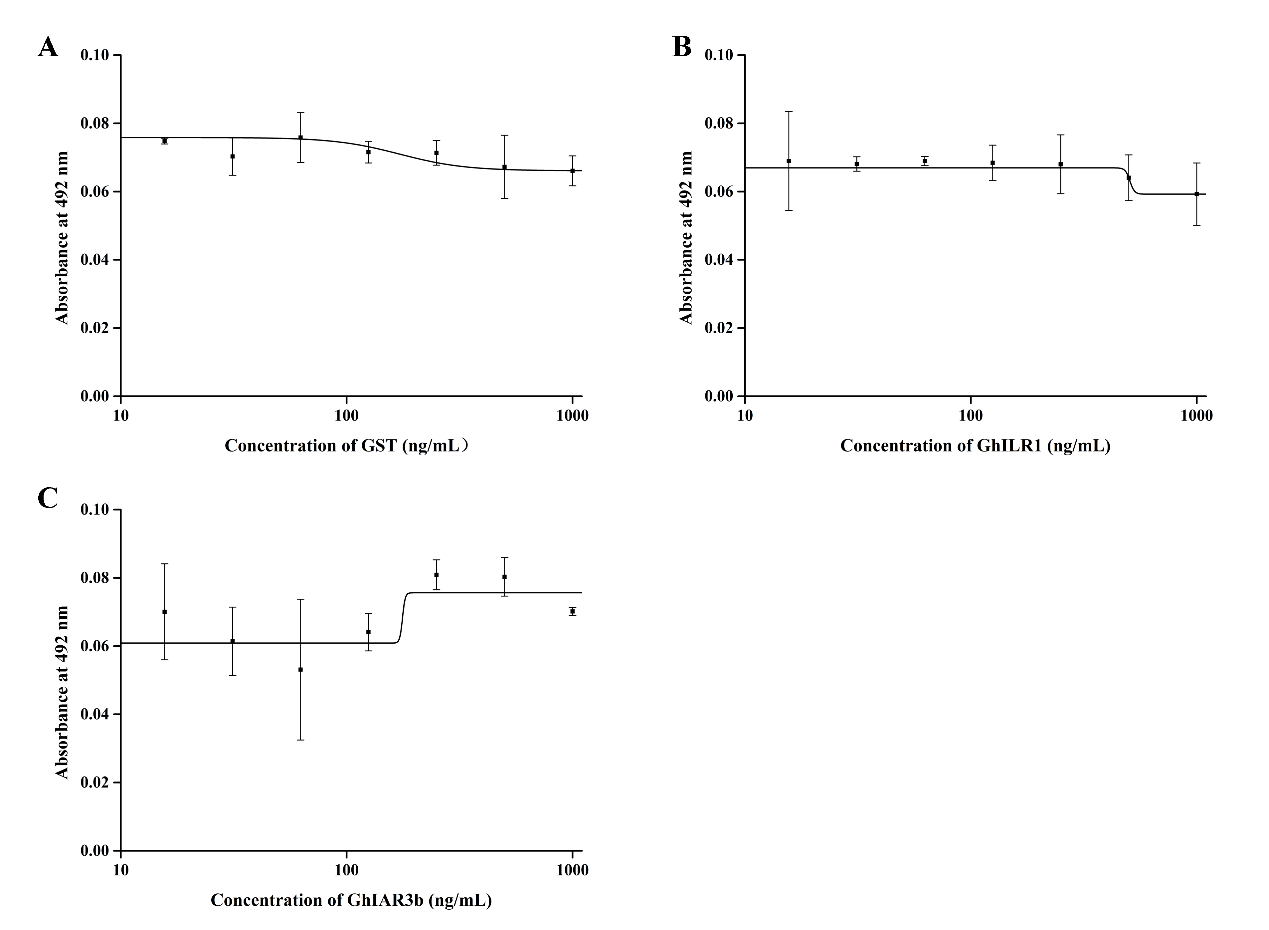


Figure S9. Cross-Reactivity of mAb 3D11 and Rabbit Antisera against to GhIAR3a with GST (A), GhILR1 (B) and GhIAR3b (C).
